# Explaining Chinese Consumers’ Continuous Consumption Intention toward Prepared Dishes: The Role of Perceived Risk and Trust

**DOI:** 10.3390/foods13010088

**Published:** 2023-12-26

**Authors:** Weihua Zhang, Jiaqiang Zheng, Yufeng Li

**Affiliations:** College of Economics and Management, Shanghai Ocean University, Shanghai 201306, China; whzhang@shou.edu.cn (W.Z.); m210701324@st.shou.edu.cn (J.Z.)

**Keywords:** prepared dishes, expectation confirmation theory, perceived risk, trust, continuous consumption intention, China

## Abstract

With the rapid development and the increasing importance of the consumer market of prepared dishes in China, it is imperative to study the formation mechanism of Chinese consumers’ continuous consumption intention (CCI) toward prepared dishes for promoting the sustainable development of Chinese prepared dishes industry. The aim of this study is to study the formation mechanism and the role of perceived risk and trust in it. Based on the Expectation Confirmation Model, this study constructed a model of continuous consumption intention of prepared dishes by introducing perceived risk and trust. 381 adult consumers were surveyed and the data was analyzed by an Exploratory Factor Analysis and the Partial Least Squares Structure Equation Model. The results showed that: (1) The confirmation of expectation had a significant positive impact on trust and satisfaction, a significant negative impact on the perceived risk (PR). Trust and satisfaction had a significant positive, while PR had a significant negative, impact on CCI. (2) The dimensions of PR included social, health, quality, psychological and purchasing risk, among which quality risk had a significant negative impact on CCI. (3) All dimensions of trust (ability, integrity, benevolence and government trust) had a significant positive impact on CCI, among which integrity trust played the most important role. (4) Overall, trust had a significant negative impact on PR. Benevolence trust could mitigate all dimensions of PR, integrity trust could mitigate all dimensions of PR except health risk, and ability trust, government trust could only mitigate quality risk. Therefore, the enterprises should pay high attention to the quality of their products and the establishment and maintenance of consumer trust, and the government should establish an authoritative image.

## 1. Introduction

With the acceleration of people’s pace of life, the prepared dishes industry has developed rapidly in China in recent years and has received strong support from the government. In 2022, China’s prepared dishes market reached 4196 billion yuan, and it is expected to exceed trillions in 2026 [1]. In 2023, the No.1 Central Document^①^ clearly proposed to ‘cultivate and develop the prepared dishes industry’. From the experience of Japan and other countries where the prepared dishes industry is well-developed, the prepared dishes industry will undergo a transformation from mainly supplying the business market to supplying the business market and consumer market evenly [2], and this transformation is also occurring in China [3]. This leads to the rapid development of prepared industry in the consumer market. Furthermore, the nature of prepared dishes as a daily goods determines that the symbol of the success of the prepared dishes industry is to obtain consumers’ continuous consumption intention (CCI) [4]. However, the consumer market of prepared dishes is currently facing the problem of consumers’ insufficient CCI. According to the data of JD.com, each prepared dishes user only purchased 1.6 times (total order number/user number) on average in 2022 [5]. It has become an imperative issue that how to make consumers integrate prepared dishes into their daily life and make it a habit to consume prepared dishes rather than just have a try.

The existing research on prepared dishes is mainly carried out from the perspective of industry and technology, and the research on the consumption intention of prepared dishes from the perspective of consumers is still relatively limited. The research from the perspective of industry focused on the whole industry [3,6] or the prepared dishes in specific regions [7] and of specific types [8]. The definition, classification, development history and development status of prepared dishes were sorted out, and the future development path was analyzed. The research from a technical perspective focused on the production [9], preservation [10] and other technologies of prepared dishes. A small amount of existing research from the perspective of consumers [1,11] analyzed the influence of individual characteristics of consumers on the cognition, attitude and consumption intention of prepared dishes, but it has not involved the issue of CCI, which is crucial to the sustainable development of the prepared dishes industry. Under the background of the rapid development of consumer market of prepared dishes, the opinions of consumers should be valued. However, the research on prepared dishes from the perspective of consumers is limited. Especially, the existing research has not given enough attention on the psychological mechanism in the transition process from consumers’ first try to the formation of CCI and the role of perceived risk and trust in it.

Therefore, based on the Expectation Confirmation Model (ECM), this study constructed a model of CCI of prepared dishes by introducing perceived risk and trust. This paper uses the model to explore the formation mechanism of consumers’ CCI toward prepared dishes. To be specific, this study mainly explores the following questions:

(1) What are the dimensions of perceived risk of prepared dishes?
(2) In the process from consumers’ first try at prepared dishes to the formation of CCI, how do consumers’ psychological factors (perceived risk, trust and satisfaction in this study) operate? And how do each dimension of perceived risk and trust operate and affect each other?

This study expands the scope of application of the ECM, reveals how psychological factors (perceived risk, trust and satisfaction) operate in the formation of CCI toward prepared dishes, which makes up for the previous research on industrial or technical perspective of prepared dishes in China, and also provides inspiration for subsequent research on the use of ECM or on the prepared dishes consumption. Meanwhile, this study can provide a theoretical basis for prepared dishes enterprises to formulate appropriate development and marketing strategies. This paper can also provide reference for the government to better fulfills its role. The findings inform how prepared dishes enterprises and the government can improve their products and/or their image according to what consumers care about, and then gain consumers’ CCI.

The remainder of this paper is organized as follows: Section 2 outlines the research hypotheses based on literature review. Section 3 describes the methodology. Section 4 presents the analysis and results. Section 5 provides a discussion of the findings and their implications. Section 6 summarizes the paper with conclusions, implications and limitations.

## 2. Theoretical Analysis

### 2.1. Expectation Confirmation Model and Perceived Risk

The Expectation Confirmation Mode is one of the most commonly used theoretical foundations in the study of continuous consumption (or use) intention. According to this theory, confirmation of expectation has a positive impact on ex post expectation and satisfaction, ex post expectation has a positive impact on satisfaction and continuous consumption (or use) intention, and satisfaction also has a positive impact on consumption (or use) intention [12]. Among them, ex post expectation is considered to be consumers’ beliefs about the product attributes after using the product [13]. Many previous empirical studies on users of shared bicycles [14], smart wearable devices [15], smartphone bank services [16] and so on have confirmed the relationship between variables in the Expectation Confirmation Model.

Under the background of information asymmetry, food enterprises have the moral hazard of using information superiority to infringe on the interests of consumers, and consumers cannot perceive and judge whether the credence-goods attributes of the food they consumed are safe. As a result, consumers will believe that there are risks in the credence-goods attributes of the food they consumed. Therefore, this paper regards perceived risk as an ex post expectation in the context of prepared dishes consumption.

Perceived risk refers to consumers’ subjective expectations of possible losses. The dimensions of consumers’ perceived risk for different goods are different, and the impact of different dimensions of perceived risk on consumer’s behavior intention is also different [17,18]. In this paper, an Exploratory Factor Analysis will be carried out to explore the dimensions of consumers’ perceived risk of prepared dishes, and the influence of each dimension of perceived risk of prepared dishes on the consumers’ CCI toward prepared dishes will also be studied.

Satisfaction is the psychological state after users’ purchase and usage experience [19], it reflects customers’ pleasure or disappoint resulting from comparing perceived performance with their expectations [20]. Therefore, positive confirmation of expectations leads to a positive effect on users’ satisfaction [21]. The same relationship should apply to prepared dishes consumption. Prepared dishes consumers will compare the actual experience of their prepared dishes use with their initial expectation. If their expectation is confirmed, they will feel satisfy with the prepared dishes. ECM argues that users’ continuance intention to use specific services is positively determined by their overall satisfaction [13]. Furthermore, many prior studies demonstrated that satisfaction is a strong determinant of continuance behavior [20,21,22]. Drawing on the ECM and previous relevant literature, we expect that satisfaction has a positive effect on consumers’ CCI towards prepared dishes. Hence, this study proposes that:

**H1:**  Consumers’ confirmation of expectation of prepared dishes has a significant positive impact on satisfaction.

**H2:**  Consumers’ satisfaction with prepared dishes has a significant positive impact on continuous consumption intention.

According to Cognitive Dissonance Theory [23], people may adjust their perception to make it consistent with the reality. Consumers’ ex post expectation can also be moderated by consumer’s experience [24]. Although consumers can only form confirmation about the search-goods attributes and experience-goods attributes (i.e., the extrinsic attributes [25], like the flavour) of prepared dishes, as long as the consumers form a positive confirmation of their expectation, they tend to believe the credence-goods attributes (i.e., the intrinsic attributes [25], like the quality) are good to avoid cognitive dissonance. Furthermore, according to the adaptation level theory, a higher ex post expectation leads to a higher level of consumer satisfaction [26]. In the context of prepared dishes consumption, the ex post expectation is represented by perceived risk. As perceived risk is a negative ex post expectation and it is demonstrated that perceived risk is negatively associated with satisfaction [27,28], we expect that the positive confirmation of expectations brought by a good consumption experience of prepared dishes will alleviate their perceived risk, and lower perceived risk of prepared dishes will strengthen consumers’ satisfaction. Hence, this study proposes that:

**H3:**  Consumers’ confirmation of expectation of prepared dishes has a significant negative impact on the perceived risk of prepared dishes.

**H4:**  Consumers’ perceived risk of prepared dishes has a significant negative impact on satisfaction.

The negative impact of perceived risk on consumption intention is widely demonstrated in the context of food consumption, especially new food like genetically modified food [29], edible insects [30], green agro-food [31] and so on. More importantly, perceived risk has a negative impact on consumers’ CCI of new food [17,32]. Additives are inevitable in the production process in prepared dishes and other processed foods. Although additives are safe as long as they are used properly and the food safety risk mainly comes from the food enterprises’ abuse of additives, consumers tend to put the blame on the additives [33]. Most Chinese consumers have a high level of perceived risk toward additives [34]. Therefore, many consumers deem processed foods like prepared dishes as unhealthy food. This may let consumers feel that eating prepared dishes may bring harmful consequences that are difficult to detect in the short term. The immaturity of prepared dishes production standards may make this situation ever worse. These factors may weaken consumers’ CCI of prepared dishes. Hence, this study proposes that:

**H5:**  Consumers’ perceived risk of prepared dishes has a significant negative impact on continuous consumption intention.

### 2.2. Trust and Perceived Risk

Information asymmetry is an important source of food safety issues and consumers’ perceived risk [35,36,37]. Under the background of information asymmetry, consumers cannot perceive and judge whether the credence-goods attributes of the food they consumed are safe. At this time, consumers will make judgments on the safety of the credence-goods attributes of food that they cannot confirm based on the credibility of the food enterprises and government regulations [38]. The result of this judgment will have an important impact on consumers’ purchasing decisions [39]. Therefore, in the context of food consumption, trust is an important tool to reduce perceived risk and facilitate transaction.

Trust is a belief that the commitment of the transaction object is credible and that the other party will fulfill its due responsibilities and obligations in the transaction relationship. Consumers’ trust in food safety mainly comes from their trust in the ability, integrity and benevolence [40]. Ensuring food safety is also an important responsibility of the government, so consumers’ trust in government regulations also plays an important role in consumers’ decision-making process of food purchasing [41,42]. Therefore, this study divides trust into ability trust, integrity trust, benevolence trust and government trust.

The quality or performance of products is one of the reason why consumers trust the enterprise [43,44]. A good consuming experience can enhance consumers’ trust [45]. After trying prepared dishes, consumers will make judgments on the search-goods attributes and experience-goods attributes of prepared dishes based on the expectations before the attempt. Positive judgment results will give consumers a good impression on the quality of prepared dishes. As a result, consumers may have a higher level of trust in the enterprises that provide prepared dishes and the government that supervises the enterprises. Hence, this study proposes that:

**H6:**  Consumers’ confirmation of expectation of prepared dishes has a significant positive impact on trust.

It is widely demonstrated that trust can reduce perceived risk [46]. In the process of new food consumption, consumers with a higher level of trust in food enterprises and government regulations show lower perceived risk [47,48]. As prepared dishes is a kind of new food, this should also apply to prepared dishes consumption. If consumers believe that the prepared dishes enterprises can and are willing to ensure the safety of prepared dishes, believe in the product information and commitments provided by the prepared dishes enterprises, and believe that the government can effectively supervise the prepared dishes enterprises, consumers will be more confident in the safety and other attributes of prepared dishes even if they cannot obtain the credence-goods attributes of prepared dishes. Hence, this study proposes that:

**H7:**  Consumers’ trust in prepared dishes enterprises and government regulations has a significant negative impact on the perceived risk of prepared dishes.

Trust plays an important role in maintaining long-term trading relationships [49]. The establishment of trust can enhance consumers’ desire to maintain existing trading relationships and enhance consumers’ CCI [50]. It is expected that consumers with a higher level of trust in prepared dishes enterprises and government regulations will be more willing to consume prepared dishes for a long time. Hence, this study proposes that:

**H8:**  Consumers’ trust in prepared dishes enterprises and government regulations has a significant positive impact on their continuous consumption intention.

## 3. Methods

### 3.1. Questionnaire

The questionnaire was divided into three parts. The first part was used to determine whether the respondents were aware of the concept of prepared dishes and whether they had purchased them. The second part is used to measure the latent variables in the research model. The continuous consumption intention was measured by items adopted from Bhattacherjee [13] and Thong et al. [51]. The satisfaction was measure by items adopted from Bhattacherjee [13] and Oliver [52]. The confirmation of expectation was measure by items adopted from Bhattacherjee [13] and Tan Chunhui et al. [53].The trust was measure by items adopted from Sun Jin et al. [54] and Yang Heng et al. [55]. The statements of some items were revised based on the context of prepared dishes consumption. This study developed a scale of perceived risk of prepared dishes based on the existing studies [17,18,56,57,58], consumer interviews and expert evaluation. Unlike common methods of measuring perceived risk, this paper directly measures consumers’ concern about the severity of the risk when measuring the perceived risk of prepared dishes, as consumers tend to be more concerned about the severity of the risk of food than the likelihood of its occurrence [59]. The items in this section were measured on a five-point Likert scale. The specific measures for each latent variable in this part is provided in Table 1.The third section is the demographic information of the respondents.

### 3.2. Participants

In order to ensure the quality of the data, Credamo, a research platform used by many authoritative journal papers [60], was used to conduct random sampling surveys. Nowadays, information dissemination and online shopping are developed, and all kinds of people may become consumers of prepared dishes. Therefore, there are no criteria like gender or region for selecting respondents.

A total of 436 questionnaires were distributed in the formal survey, and 381 valid questionnaires were collected after excluding invalid questionnaires, accounting for 87.4%. The characteristics of the respondents are basically in line with the consumer portrait of prepared dishes [11], which is a good representativeness. Table 2 shows the basic information of the respondents:

## 4. Results

### 4.1. The Dimensions of the Perceived Risk of Prepared Dishes

An Exploratory Factor Analysis was used to explore the dimensions of perceived risk of prepared dishes. The principal component analysis method was selected, and based on the standard extraction factors with eigenvalues greater than 1 and loads greater than 0.5, the rotation method was selected as the maximum variance method. Items FX2, FX5, and FX15 with low or double loads in the initial analysis were deleted one by one according to the load, and the exploratory factor analysis was re-performed after each deletion. The total variance explanation matrix after removing the items showed that the explanatory power of the 5-factor model for the total variance was 70.76%, indicating that the five factors could fully express the information of the measured items.

The rotation component matrix is shown in Table 3. Factor 1 contains measurement items that are mainly related to consumers’ concerns about others’ perceptions of consuming prepared dishes, so it is named social risk. Factor 2 is mainly related to the possible impact of prepared dishes on physical health, so it is named health risk. Factor 3 is mainly related to consumers’ concern about the quality of prepared dishes, so it is named quality risk. Factor 4 is mainly related to the possible impact of prepared dishes on mental health, so it is named psychological risk. Factor 5 is mainly related to the purchasing and return of prepared dishes, so it is named as purchasing risk. The above factors can explain 15.89%, 15.21%, 15.06%, 14.03% and 10.57% of the total variance, respectively.

**Figure 1:**
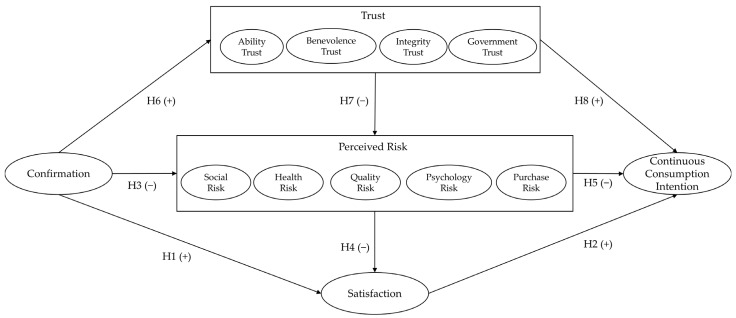
Conceptual model.

Based on the analysis in the theoretical analysis part and the result of the exploratory factor analysis of perceived risk, the complete model, including all dimensions of trust and perceived risk, of continuous consumption intention of prepared dishes is constructed, as shown in Figure 1.

### 4.2. Results of Measurement Model

Before evaluating the structural model, the measurement model’s reliability and validity need to be examined. This study conducted the Confirmatory Factor Analysis by the software SmartPLS. As suggested by prior studies [61], this study measured the reliability of the scale by the Cronbach’s alpha (α), measured the convergent validity by the composite reliability (CR) and the average variance extracted (AVE). Perceived risk and trust were measured using the average value of the first-order measurement model item measurements. During the analysis, the measurement items of confirmation of expectation, ability trust, and government trust were adjusted to improve the reliability and validity. According to the analysis results in Table 4, of each latent variable, the α was greater than 0.6, the CR was greater than 0.8, and the AVE was greater than 0.5, indicating that the reliability and convergence validity of the scale were good [62].

The discriminant validity indicates how much the measure is adequately distinguishable from related constructs. The Fornell and Larcker criterion is the most popular method for testing discriminant validity [63] and has been used in many studies of different areas [64,65]. Therefore, this study also adopted the Fornell and Larcker criterion to test the discriminant validity. As per the Fornell and Larcker criterion, if the square root of AVE in each latent variable in the correlation matrix are bigger than the correlation coefficient of latent variables, the discriminant validity is constructed [66]. According to the analysis results in Table 5, the discriminant validity of the present study met the Fornell–Larcker criterion since the square root of AVE (numbers on diagonal) is larger than the correlation coefficient values of the two variables (numbers off-diagonal).

### 4.3. Control and Test of Common Method Biases

In order to reduce the deviation of the common method as much as possible, the procedural ex-ante control measures adopted in this paper [67] include: (1) developing questionnaires with reference to domestic and foreign maturity scales, and improving the scientificity of the expression of items as much as possible through expert review and pre-investigation. (2) Ensure that respondents are not repeatedly surveyed and the anonymity of respondents through the Credamo platform. In addition, the results of Harman’s one-way test showed that there were 8 factors with eigenroots greater than 1, and the variance explanation rate of the largest factor was 38.90%, which was less than 50% [68]. Therefore, there is no serious common method bias in this paper.

### 4.4. Structural Model Evaluation

This study is an exploratory research on prepared dishes to a certain extent and the Partial Least Squares Structural Equation Modeling (PLS-SEM) is more applicable to exploratory theoretical models [69]. Compared with Covariance Based Structural Equation Modeling (CB-SEM), PLS-SEM is equally effective, provides almost similar results [70], but requires less samples, [71] has no restrictive assumptions about data distribution, and is more applicable to complex models [72,73]. Thus, PLS-SEM was deemed appropriate for the research context. Therefore, this study used the software SmartPLS that is based on PLS-SEM method to test the research hypothesis.

Adopting the tests used for goodness of fit from previous research [74], this study also utilized the Standardized Root Mean Square Residual (SRMR) and R square to examine the fitness of the proposed model. SRMR is a measure of the average difference between the observed and model-implied correlations [75]. According to Demler et al. [76], SRMR values range from 0 to 1, where lower values indicate a better fit, but the specific thresholds may vary depending on the context and the intricacy of the model [77]. The acceptable range of SRMR is <0.10 [75]. R square represents the proportion of variation in the dependent variable that can be explained by the independent variable [78]. According to Henseler et al. [79], 0.33–0.67 is a acceptable range of R square. In this study, the SRMR is 0.09, the R square is 0.449–0.644 (CCI = 0.644, PR = 0.468, Sat = 0.458, Trust = 0.449), all falling within the acceptable range. Those indicated a good fit for the model in this study.

In the SmartPLS software, PLS-SEM algorithm and Bootstrapping re-sampling method were used to measure the path coefficients between variables and their significance. In the path analysis, the perceived risk of prepared dishes and trust were measured by the average value of the measurement results of the corresponding first-order measurement model. According to the test results Table 6: (1) Confirmation of expectation has a significant positive impact on trust (β=0.670) and satisfaction (β=0.543), and has a significant negative impact on the perceived risk of prepared dishes (β=−0.158) (Hypothesis H1, H3 and H6 are supported). (2) Trust (β=0.173) and satisfaction (β=0.550) have a significant positive impact on continuous consumption intention, and perceived risk of prepared dishes (β=−0.185) has a significant negative impact on continuous consumption intention (Hypothesis H2, H5 and H8 are supported). (3) Trust (β=−0.568) has a significant negative impact on perceived risk of prepared dishes (Hypothesis H7 is supported). (4) The perceived risk of pre-cooked food has a significant negative impact on satisfaction (β=−0.205) (Hypothesis H4 is supported).

### 4.5. The Influence of Each Dimension of Trust on Each Dimension of Perceived Risk of Prepared Dishes

To explore the influence of each dimension of trust on the perceived risk of prepared dishes, this paper proposes the corresponding sub-hypotheses on the basis of existing research.

**H7a–t:**  ability trust, integrity trust, benevolence trust and government trust respectively have a significant negative effects on social risk, health risk, quality risk, psychological risk and purchasing risk.

The same method is used to test the above sub-hypotheses. As is shown in the test results Table 7: (1) Ability trust has a significant negative impact on quality risk, a significant positive impact on health risk, but has no significant impact on other risks (Sub-hypothesis H7c is supported, H7a–b and H7d–e are not). (2) Integrity trust has a significant negative impact on each risk other than health risk (Sub-hypotheses H7f and H7h–j are supported, H7g is not). (3) Benevolence trust has a significant negative impact on each dimension of the perceived risk of prepared dishes (Sub-hypothesis H7k–o are supported). (4) Government trust has a significant negative impact on only quality risk (Sub-hypothesis H7r is supported, H7p–q and H7s–t are not).

### 4.6. The Influence of Each Dimension of Perceived Risk and Trust on Continuous Consumption Intention

To explore the impact of various dimensions of perceived risk of prepared dishes and trust on the continuous consumption intention of prepared dishes, this paper proposes the corresponding sub-hypotheses on the basis of existing research.

**H5a–e:**  social risk, health risk, quality risk, psychological risk and purchasing risk respectively have a significant negative impact on the continuous consumption intention of prepared dishes.

**H8a–d:**  Ability trust, integrity trust, benevolence trust and government trust respectively have a significant positive impact on the continuous consumption intention of prepared dishes.

The same method is used to test the above sub-hypotheses. As is shown in the test results Table 8: (1) Among the five dimensions of perceived risk of prepared dishes, only quality risk has a significant negative impact on the continuous consumption intention of prepared dishes (Sub-hypothesis H5c is supported, and the other sub-hypotheses are not). (2) Each dimension of trust has a significant positive impact on the continuous consumption intention of prepared dishes, and the path coefficient of integrity trust is the largest (Sub-hypothesis H8a–d are supported).

## 5. Discussion

The purpose of the current study was to cast light on the role of psychological factors, perceived risk, trust and satisfaction especially, in the formation of the continuous consumption intention (CCI) of prepared dishes and the formation mechanism. According to the results, this study found that:

The relationships between concepts in the Expectation Confirmation Model (ECM), especially the effect of the ex post expectation (represented by perceived risk in this study) on the CCI, was demonstrated in this study. This is not only consistent with the ECM itself [12], but also consistent with many existing empirical studies basing the ECM [15]. Our findings indicate that consumers’ confirmation of the search-goods attributes and experience-goods attributes of prepared dishes can enhance consumers’ satisfaction, enhance consumers’ trust in prepared dishes enterprises and the government regulations, and reduce the perceived risk of prepared dishes caused by consumers’ inability to perceive the credence-goods attributes of prepared dishes. As a result, a higher level of consumers’ satisfaction and trust, and a lower level of perceived risk are helpful to improve consumers’ CCI toward prepared dishes. Furthermore, our finding that a lower level of perceived risk led to a higher level of satisfaction is in consistent with the ECM and the adaptation level theory.

Via the exploratory factor analysis, this study found that the dimensions of perceived risk of prepared dishes include social risk, health risk, quality risk, psychological risk and purchasing risk. The dimensions are different from the dimensions of perceived risk of other goods or services [80], as it should be. Furthermore, this study also found that the effect of different dimensions of perceived risk of prepared dishes on continuous consumption intention toward prepared dishes differ. This is similar with the findings of the existing studies on internet shopping behavior [81]. To be specific, the quality risk had a negative impact on the CCI while other dimensions didn’t have significant impact. Our findings indicate that Chinese consumers have many concerns about prepared dishes, and the quality is the main concern when Chinese consumers face the choice of whether or not to continue consuming prepared dishes.

The role of trust in reducing perceived risk of prepared dishes and increasing the CCI toward prepared dishes was confirmed in this study. This is similar with the prior studies in other contexts [82,83]. It indicates that consumers’ trust in product providers and regulators can reduce consumers’ perceived risks of the product itself and increase their CCI. To be specific, our findings indicate that consumers will have a lower level of perceived risk and be more willing to continue consuming prepared dishes if they believe that prepared dishes enterprises have the ability to produce high-quality prepared dishes, provide consumers with authentic and reliable relevant information and are willing to protect the interests of consumers.

This study also found that the different dimensions of trust played different roles in the two relationships above. From the perspective of reducing the perceived risk of prepared dishes, consumers believe that benevolent prepared dishes enterprises will let them bear the least risk; honest prepared dishes enterprises can ensure the quality of prepared dishes and related services, but the prepared dishes they produce is not necessarily healthy; capable prepared dishes enterprises can only ensure the safety of prepared dishes, but they may abuse their capabilities to produce unhealthy prepared dishes; the main role of government regulations is to ensure the quality and safety of prepared dishes. From the perspective of improving consumers’ CCI toward prepared dishes, consumers most hope that prepared dishes enterprises can treat consumers honestly and provide consumers with credible decision-making basis.

## 6. Conclusions and Implications

### 6.1. Conclusions

This study aimed to explore the psychological mechanism in the transition process of consumers’ consumption behavior from the initial try to continuous consumption intention and the role of perceived risk and trust in it. Our findings showed that all hypothesis based on the ECM (the ex post expectation was represented by perceived risk in this study) and the role of trust were demonstrated by empirical analysis. This verified the applicability of the ECM itself and in the context of prepared dishes consumption. To some extent, this is consistent with the connotation of the Stimulus-Organism-Response (SOR) model, which in turn reflects the scientific nature of this study to a degree.

Through the exploratory factor analysis, we found that the dimensions of perceived risk of prepared dishes included social, health, quality, psychological and purchasing risk. By testing of sub-hypothesis, we found that only the quality risk is negatively associated with consumers’ CCI. This study demonstrated again that the dimension of consumers’ perceived risk of different goods or services and the effect of different dimensions on consumers’ behavior or behavior intention vary. This fact and our findings highlight and justify the necessity of our study.

Overall, trust can mitigate consumers’ perceived risk and improve CCI. Meanwhile, each dimension of trust has a positive effect on CCI with different importance and their effects on perceived risk vary. This provides a new direction for the marketing work of the prepared dishes enterprises. That is, the enterprises should attach great importance to the construction of their own image. To be specific, they should demonstrate their ability, integrity and benevolence to consumers. In addition, the results of this study also indicate that the government should play an important role in promoting the development of the prepared vegetable industry.

### 6.2. Theoretical Implications

This study makes several theoretical contributions. First, this study determined the dimensions of perceived risk of prepared dishes. By exploring the dimensions, it not only deepens the understanding of Chinese consumers’ perceived risk of prepared dishes, but also might be helpful to the future related researches regarding the consumption of prepared dishes. Second, by applying the ECM, this study expands the scope of application of the expectation confirmation model. To some extent, this study also demonstrates the applicability of a modified ECM in the context of prepared dishes consumption. Finally, this study fills a research gap by focusing on the issues of consumers’ CCI of prepared dishes and extends the existing literature on the consumption of prepared dishes in China.

### 6.3. Practical Implications

The empirical results have positive management implications and policy implications for the sustainable development of prepared dishes industry in China.

The management implications for the prepared dishes enterprises are as follows: First, since this study found that confirmation of expectation can mitigate perceived risk, improve consumers’ trust and satisfaction, the enterprises should try their best to raise the level of consumers’ confirmation of expectations toward prepared dishes. To do this, the enterprises should avoid letting consumers be disappointed due to too high expectations generated from exaggerated or false publicity. Meanwhile, the enterprises should strive to give consumers a better consumption experience by improving the flavor, mouthfeel and so on.

Second, as this study found, perceived risk, quality risk specifically, is negatively related to consumers’ CCI and satisfaction. To reduce the consumers’ perceived risk of prepared dishes, the enterprises should strengthen their quality management system to effectively ensure the quality of prepared dishes. Meanwhile, since perceived risk is a subjective cognition, the enterprises may use diverse marketing devices to improve consumers’ perception of prepared dishes.

Third, in order to get consumers’ CCI, enterprises should pay attention to get consumers’ trust. Apart from raising the level of confirmation of expectations, the enterprises also should strengthen the transparency of their production and operation process, strictly implement the commitment to consumers, so as to establish an integrity image. At the same time, the enterprises should proactively show consumers their production qualifications, certifications of authoritative organizations and so on to testify their abilities. In addition, the enterprises should also actively assume social responsibility and strengthen communication with consumers to show their benevolence.

Finally, the enterprises should also pay attention to the packings, logistics and so on to make consumers more satisfied.

The policy implication for the government is that it should perfect the laws, regulations and regulatory system for the supervision of prepared dishes, and release authoritative and fair regulatory information timely, so as to establish an authoritative image.

## 7. Limitations and Future Research

This study contributes to the literature on the ECM and the understanding of Chinese consumers’ consumption of prepared dishes. This study provides practical guidance for prepared dishes enterprises and governments. With the significant results, this study still has some limitations. Firstly, this study only examines the role of perceived risk and trust in the translation process of consumers’ first try at prepared dishes to the formation of consumers’ CCI, but there should be other influencing psychological factors, and the role of food neophobia could be considered in the future. Second, since this study mainly focus on the translation process and the role of psychological factors in it, we measure the overall evaluation of consumers on prepared dishes with confirmation of expectation. In the future, consumers’ evaluation of prepared dishes can be divided into different dimensions to determine which search-goods and experience-goods attributes of prepared dishes consumers value more.

①No.1 Central Document is the first policy document issued by the Central Committee of the Communist Party of China and the State Council every year. This policy document is of great significance for China’s agricultural development.

## Figures and Tables

**Table 1:** Latent variables and measurement.

Latent Variables	Items	Measurement
Continuous Consumption Intention	CCI1	I intend to continue buying prepared dishes in the future.
	CCI2	I would like to recommend prepared dishes to my friends.
	CCI3	Compared with traditional ways of cooking meals, I prefer to use prepared dishes to cook meals.
	CCI4	If I could, I would still buy prepared dishes to cook meals.
Satisfactioin	Sat1	I think it is a wise choice to use prepared dishes in cooking meals.
	Sat2	I feel pleased about my experience of using prepared dishes.
	Sat3	I feel satisfied about my experience of using prepared dishes.
	Sat4	Overall, prepared dishes are satisfactory.
Confirmation	Con1	The performance of prepared dishes is better than what I expected.
	Con2	Prepared dishes are more convenient than I expected.
	Con3	The cost-performance of prepared dishes is better than what I expected.
	Con4	Overall, most of my expectations from using prepared dishes are confirmed.
Perceived risk	FX1	Prepared dishes are made from unqualified materials.
	FX2	The nutritions in prepared dishes are not balanced.
	FX3	There are illegal or excessive additives in prepared dishes.
	FX4	The packing of prepared dishes will produce harmful substances.
	FX5	It is difficult to ensure the transportation conditions (such as refrigeration, freezing, etc.) during the transportation of prepared dishes.
	FX6	It takes me long time buying the suitable prepared dishes.
	FX7	The logistics time will be long if I buy prepared dishes online.
	FX8	It will take me long time if I need returen or exchange prepared dishes.
	FX9	Long-term consumption of prepared dishes will cause damage to my health.
	FX10	Prepared dishes with quality problems will damage my health.
	FX11	Certain prepared dishes of nuknown brands can be harmful to my health.
	FX12	I am concerned about the health risk associated with prepared dishes.
	FX13	Prepared dishes are closely related to health, so I have to be very careful when purchasing prepared dishes.
	FX14	Cooking with prepared dishes over the long term increases my food expenses compared to traditional methods.
	FX15	The monetary cost of returning prepared dishes is high in case I bought faulty prepared dishes.
	FX16	Eating prepared dishes with quality problems will make me ill and therefore increase my medical expenses.
	FX17	Prices for prepared dishes are unstable and fluctuate widely.
	FX18	Having purchased unqualified prepared dishes makes me anxious.
	FX19	It makes me irritated to get into trouble with the seller after purchasing faulty prepared food.
	FX20	Purchasing faulty prepared dishes causes psychological damage and frustration to me.
	FX21	My family won’t like the prepared dishes I buy.
	FX22	My family will think it’s unwise for me to buy prepared dishes.
	FX23	My family will think I’m lazy for buying prepared dishes.
	FX24	My family will think that I am being irresponsible to my health by buying prepared dishes.
Ability Trust	AT1	I believe that prepared food enterprices have the ability and resources to provide high-quality prepared dishes.
	AT2	I believe that prepared dishes enterprises have the ability and resources to meet consumer demands and preferences for prepared dishes.
	AT3	I believe that prepared dishes enterprises have the ability and resources to fulfill their promises to consumers.
Benevolence Trust	BT1	I believe that prepared dishes enterprises would act in my best interest.
	BT2	If I required help, prepared dishes enterprises would do its best to help me.
	BT3	I believe that prepared dishes enterprises is interested in my well-being, not jutst its own.
Integrity Trust	IT1	I believe that prepared dishes enterprises are hones to customers.
	IT2	I believe that the informations provided by prepared dishes enterprises are true.
	IT3	I believe that prepared dishes enterprises will keep their promises.
Government Trust	GT1	I believe that the government has the ability to ensure the safety of prepared dishes.
	GT2	I believe that the government has sufficient knowledge of ensuring the safety of prepared dishes.
	GT3	I believe that the government is honest about the safety issues of prepared dishes.
	GT4	I believe that the government is sufficiently open with the safety issues of prepared dishes.
	GT5	I believe that the government gives special attention to the safety issues of prepared dishes.
	GT6	I believe that the government is doing a good job in ensuring the safety of prepared dishes.

**Table 2:** Sample description.

Variable	Category	Frequency	Percentage (%)
Gender	Male	183	48
	Female	198	52
Age	18–25	118	31
	26–35	177	46.5
	36–45	56	14.7
	46–55	26	6.8
	More than 60	4	1
Education	Junior high school or below	6	1.6
	Senior high school	6	1.6
	College	20	5.2
	Bachelor’s degree	278	73
	Master’s degree or above	71	18.6
Monthly Income	<3000	66	17.3
	3001–5000	49	12.9
	5001–8000	101	26.5
	8001–10,000	66	17.3
	>10,001	99	26
Marital Status	Single	156	40.9
	Married	225	59.1
The presence of child or elder	Yes	77	20.2
	No	304	79.8

**Table 3:** The rotated component matrix-EFA results.

Item	Factors and Scale Items					Name
	1	2	3	4	5	
FX21	0.887					Social risk (FX1)
FX22	0.881					
FX23	0.791					
FX24	0.772					
FX11		0.746				Health risk (FX2)
FX13		0.728				
FX10		0.715				
FX16		0.679				
FX12		0.593				
FX3			0.794			Quality risk (FX3)
FX4			0.778			
FX1			0.766			
FX9			0.693			
FX18				0.804		Psychological risk (FX4)
FX20				0.801		
FX19				0.775		
FX17				0.532		
FX6					0.715	Purchasing risk (FX5)
FX14					0.638	
FX7					0.616	
FX8					0.592	

Extraction method: principal component. Rotating method: Orthogonal rotating method with Kaiser standardization. a. Rotation converges after 7 iterations.

**Table 4:** Analysis results of reliability and convergence validity.

Latent Variable	α	CR	AVE	Construct	α	CR	AVE
Confirmation (Con)	0.705	0.871	0.772	Continuous Consumption Intention (CCI)	0.765	0.851	0.588
Perceived Risk (PR)	0.852	0.894	0.629	Satisfaction (Sat)	0.768	0.852	0.591
Social Risk (SR)	0.938	0.956	0.844	Trust (Trust)	0.886	0.921	0.745
Health Risk (HR)	0.854	0.895	0.63	Ability Trust (AT)	0.623	0.841	0.726
Quality Risk (QR)	0.844	0.895	0.681	Integrity Trust (IT)	0.771	0.867	0.686
Psychological Risk (PsR)	0.846	0.899	0.694	Benevolence Trust (BT)	0.812	0.889	0.727
Purchasing Risk (PuR)	0.795	0.869	0.628	Government Trust (GT)	0.726	0.829	0.549

**Table 5:** Analysis results of discriminant validity.

	1	2	3	4	5	6	7	8	9	10	11	12	13	14
1 IT	0.924													
2 CCI	0.617	0.876												
3 PR	−0.597	−0.529	0.891											
4 SR	−0.577	−0.485	0.818	0.959										
5 HR	−0.250	−0.257	0.729	0.418	0.891									
6 QR	−0.695	−0.652	0.800	0.652	0.407	0.908								
7 PsR	−0.325	−0.264	0.786	0.463	0.684	0.475	0.913							
8 PuR	−0.479	−0.407	0.840	0.672	0.484	0.594	0.558	0.890						
9 Sat	0.630	0.763	−0.449	−0.460	−0.162	−0.581	−0.238	−0.303	0.877					
10 AT	0.692	0.574	−0.441	−0.416	−0.115	−0.623	−0.225	−0.325	0.622	0.923				
11 Con	0.621	0.611	−0.508	−0.399	−0.282	−0.599	−0.323	−0.393	0.654	0.565	0.938			
12 BT	0.765	0.593	−0.612	−0.603	−0.291	−0.646	−0.334	−0.518	0.573	0.612	0.588	0.910		
13 Trust	0.898	0.680	−0.615	−0.584	−0.238	−0.743	−0.331	−0.498	0.696	0.849	0.670	0.874	0.929	
14 GT	0.635	0.564	−0.463	−0.410	−0.154	−0.598	−0.249	−0.387	0.584	0.643	0.536	0.634	0.837	0.861

Note: the value on the diagonal is the square root of the corresponding AVE value, and the value on the non-diagonal is the correlation coefficient between variables.

**Table 6:** The results of hypothesis testing.

Path			Coefficient	S.E.	T	*p*	Result
Confirmation	→	Trust	0.670	0.048	13.918	0.000	Yes
Confirmation	→	Perceived Risk	−0.158	0.056	2.825	0.005	Yes
Confirmation	→	Satisfaction	0.543	0.061	8.892	0.000	Yes
Trust	→	Continuous Consumption Intention	0.173	0.065	2.657	0.008	Yes
Perceived Risk	→	Continuous Consumption Intention	−0.185	0.053	3.497	0.000	Yes
Satisfaction	→	Continuous Consumption Intention	0.550	0.059	9.290	0.000	Yes
Trust	→	Perceived Risk	−0.568	0.045	12.660	0.000	Yes
Perceived Risk	→	Satisfaction	−0.205	0.045	4.537	0.000	Yes

Note: The significance level is α = 0.05. The same applies hereinafter.

**Table 7:** The results of sub-hypothesis testing.

Path			Coefficients	*p*	Result	Path			Coefficients	*p*	Result
Ability Trust	→	Social Risk	0.031	0.656	No	Benevolence Trust	→	Social Risk	−0.399	0.000	Yes
	→	Health Risk	0.158	0.046	No		→	Health Risk	−0.281	0.000	Yes
	→	Quality Risk	−0.196	0.001	Yes		→	Quality Risk	−0.164	0.023	Yes
	→	Psychological Risk	0.048	0.528	No		→	Psychological Risk	−0.199	0.010	Yes
	→	Purchasing Risk	0.108	0.167	No		→	Purchasing Risk	−0.350	0.000	Yes
Integrity Trust	→	Social Risk	−0.297	0.000	Yes	Government Trust	→	Social Risk	0.002	0.977	No
	→	Health Risk	−0.157	0.057	No		→	Health Risk	−0.020	0.776	No
	→	Quality Risk	−0.335	0.000	Yes		→	Quality Risk	−0.174	0.004	Yes
	→	Psychological Risk	−0.182	0.031	Yes		→	Psychological Risk	−0.059	0.350	No
	→	Purchasing Risk	−0.229	0.019	Yes		→	Purchasing Risk	−0.099	0.147	No

**Table 8:** The results of sub-hypothesis testing.

Path			Coefficient	*p*	Result	Path			Coefficient	*p*	Result
Social Risk	→	Continuous Consumption Intention	−0.112	0.125	No	Ability Trust	→	Continuous Consumption Intention	0.182	0.017	Yes
Health Risk	→		−0.073	0.233	No	Integrity Trust	→		0.231	0.015	Yes
Quality Risk	→		−0.587	0.000	Yes	Benevolence Trust	→		0.190	0.016	Yes
Psychological Risk	→		0.128	0.067	No	Government Trust	→		0.182	0.020	Yes
Purchasing Risk	→		−0.041	0.586	No						

## Data Availability

The data that support the findings of this study are available from the corresponding authors upon reasonable request.
